# Source-Only Transportability of Engineered ECG Features for Healthy-Versus-Myocardial Infarction Classification

**DOI:** 10.3390/diagnostics16132061

**Published:** 2026-07-01

**Authors:** Fatih Aydın, Sefer Usta, Ezgi Kalaycıoğlu, Onder Aydemir

**Affiliations:** 1 Department of Electrical and Electronics Engineering, Karadeniz Technical University, 61080 Trabzon, Türkiye; aydinf@ktu.edu.tr; 2 Ahi Evren Thoracic and Cardiovascular Surgery Training and Research Hospital, Trabzon University, 61335 Trabzon, Türkiye; seferusta2000@yahoo.com (S.U.); ezgikalay@gmail.com (E.K.); 3 METAM (Medical Device Design and Production Application and Research Center), Karadeniz Technical University, 61080 Trabzon, Türkiye

**Keywords:** electrocardiography, myocardial infarction, machine learning, PTB-XL, external validation, dataset shift, engineered ECG features, transportability, decision support

## Abstract

**Background/Objectives:** Electrocardiogram (ECG)-based myocardial infarction (MI) classifiers may achieve high internal validation performance but show reduced performance when applied to data from another source. The task is a controlled binary healthy-versus-MI benchmark and is not intended to represent real-world chest-pain triage or autonomous clinical deployment. This study evaluated the source-only transportability of engineered 12-lead ECG feature families for binary healthy-versus-MI classification across a cardiologist-annotated hospital dataset and PTB-XL. **Methods:** The hospital dataset contained 1749 usable recordings from 1434 patients after excluding 206 broken-data records, with 1550 Healthy and 199 MI recordings. The matched PTB-XL binary subset contained 14,982 recordings from 13,436 patients, with 9513 Healthy and 5469 MI recordings. Eleven engineered feature families and five classifier families were compared under preprocessing, patient-aware splitting, source-validation hyperparameter and threshold selection, and bootstrap uncertainty estimation. The reported leading rows are the highest observed configurations in a prespecified benchmark grid, not locked clinical models. **Results:** Internal performance was higher than strict source-only transfer performance. In the hospital dataset, fiducial interval descriptors with Extra Trees reached balanced accuracy 0.775 and receiver operating characteristic area under the curve (ROC-AUC) 0.855. In PTB-XL, a broad hybrid feature bank with ST-segment information and XGBoost reached a balanced accuracy of 0.898 and ROC-AUC of 0.965. Strict source-only transfer was weaker and asymmetric: the highest observed balanced accuracy was 0.580 for hospital-to-PTB-XL transfer and 0.632 for PTB-XL-to-hospital transfer. Ranking transportability and operating-threshold transportability diverged, most notably for hospital-to-PTB-XL transfer, where ROC-AUC was 0.774 but sensitivity at the source-selected threshold was only 0.164. A secondary target-threshold analysis improved balanced accuracy to 0.682 and 0.640, respectively, but this used target labels only to re-select the operating threshold and was not a strict source-only result. **Conclusions:** The findings indicate a transportability gap: PTB-XL-to-hospital transfer was more balanced than hospital-to-PTB-XL transfer, but neither direction achieved performance comparable to internal validation. The source-only operating-point results are not acceptable for clinical MI screening or decision support without additional calibration, target-setting validation, and prospective assessment.

## 1. Introduction

Electrocardiography (ECG) is one of the first tests used when myocardial infarction (MI) or acute coronary syndrome is suspected because it is fast, low-cost, and available before many other diagnostic steps. An ECG signal consists of characteristic waveforms and intervals that reflect different phases of cardiac electrical conduction and recovery. In general, the P wave represents atrial depolarization, the QRS complex reflects ventricular depolarization, and the T wave corresponds to ventricular repolarization. Clinical MI definitions and acute coronary syndrome guidelines rely on ECG findings such as ST-segment deviation, T-wave changes, and other ischemia-related waveform abnormalities for early detection and risk assessment [1,2,3]. Repolarization changes also remain central when ischemia-related electrical instability is discussed [4].

Artificial intelligence (AI) applications in ECG-based MI assessment have shown substantial diagnostic potential. Recent studies have reported strong results for task-specific ECG applications related to ST-elevation MI (STEMI) or occlusion MI. Zhao et al. trained an AI classification system using a dataset with 667 STEMI ECGs and 7571 control ECGs and reported an external area under the curve (AUC) of 0.9954, sensitivity of 96.75%, and specificity of 99.20% [5]. In another study, Lee et al. used 18697 ECGs, including 1745 STEMI cases, and reported a 92.1% accuracy, 95.4% sensitivity, and 91.8% specificity [6]. Al-Zaiti et al. developed and externally validated occlusion-MI models on 7313 consecutive patients from multiple clinical sites [7]. Wu et al. reported an external AUC of 0.93 for a least absolute shrinkage and selection operator (LASSO)-based STEMI model [8]. ECG-based AI has also been extended beyond acute labeling to remote ambulance support, culprit-vessel analysis, post-STEMI left ventricular dysfunction, and mortality prediction [9,10,11,12]. Although these studies show the potential of ECG-based AI in focused STEMI or occlusion-MI settings, they do not isolate the source-only transportability of fixed engineered features across datasets.

Classical engineered ECG features remain useful because they connect model inputs to clinically interpretable ECG structures, including waveform morphology, fiducial intervals, ST-segment behavior, T-wave and repolarization patterns, and multi-lead relationships. Prior studies that used engineered features have shown strong performance in defined MI-detection settings, including morphology and energy-entropy descriptors with 92.69% inter-patient accuracy [13], lead-specific optimal features with an AUC of 0.99 [14], single-lead and nonlinear ECG descriptors [15,16], morphology-based MI localization [17], ischemia-related time-frequency features [18], three-dimensional STEMI display support [19], and dynamic ECG feature extraction with 94.75% accuracy on PTB and 84.96% accuracy on an independent clinical dataset [20]. Although these strong performances show the potential of engineered features, they also motivate the question of transportability across datasets because engineered descriptors can depend on acquisition conditions, preprocessing, and the signal characteristics of the source ECGs.

Large-scale ECG-AI studies have shown that neural networks can learn clinically useful patterns from 12-lead ECG signals and ECG images, which is important for real-world use when raw digital ECG signals are not always available [21,22]. More recent MI-focused studies have moved toward practical deployment issues, including lead-aware modeling, paper-ECG digitization, and transfer across datasets. Liu et al. proposed EvoMBN, a genetic-algorithm-optimized multi-branch network with a lead squeeze-and-excitation mechanism, and reported 90.80% accuracy for MI detection on PTB-XL after transferring architecture information learned from PTB [23]. Rai et al. studied paper-ECG digitization and showed that retraining with PTB-XL, public ECG images, and hospital paper-ECG data improved real-hospital performance, with the final hospital MI model reaching an approximately 92.55% accuracy [24]. Bao et al. combined lightweight MI detection, Shapley additive explanations (SHAP)-based lead selection, and transfer learning from PTB-XL to a clinical dataset, reporting strong PTB-XL performance and high accuracy after transfer learning on the proprietary CPC dataset [25]. Guo et al. further showed that clinically guided lead grouping can improve MI localization on PTB-XL, with LG-MSL achieving an AUC of 0.9546 and micro-F1 of 0.8656 [26]. Together, transfer studies show that dataset differences, lead information, and deployment conditions remain central challenges in ECG-based MI detection. However, these solutions generally rely on target-domain data, deep architectures, architecture transfer, retraining, fine-tuning, or lead selection, which can increase computational cost and model complexity.

The methodological motivation of this study is that strong internal validation performance does not necessarily imply external transportability. Internal validation estimates performance when model development and testing occur within the same data source, whereas external validation evaluates whether a model remains valid in data from a different source or setting [27,28]. Diagnostic-AI reporting and risk-of-bias guidance similarly emphasize the need to describe the data source, participant spectrum, predictors, outcome definitions, and intended-use setting when interpreting model performance [29,30,31]. Dataset shift can reduce machine learning performance even when the nominal signal type and endpoint appear similar because changes in acquisition hardware, patient spectrum, labeling practice, prevalence, preprocessing, or selected operating threshold can alter the relationship between features and labels [32,33]. PTB-XL is a widely used public benchmark because it provides 21,837 12-lead ECG records from 18,885 patients with structured diagnostic labels and suggested folds for development and testing [34,35]. However, strong performance inside PTB-XL or inside a single hospital source does not by itself establish that the same feature representation and source-selected threshold will remain valid in another setting. The term transportability is used here in the empirical diagnostic-model sense of whether a source-trained feature extraction, classifier, and threshold-selection pipeline retains useful performance in a different ECG data source without target-domain adaptation. This usage is related to, but narrower than, formal causal transportability theory, which defines graphical and statistical conditions under which relations can be transported between populations [36]. Therefore, transportability denotes source-only external validity of a predictive pipeline, not a proof of causal transportability.

Consistent with this methodological focus, the present work is an ECG-methodology benchmark rather than a real-world chest-pain triage study. The binary healthy-versus-MI design deliberately excludes other common ECG abnormalities, including ischemia without an MI label, arrhythmias, conduction disease, and non-MI structural or repolarization abnormalities; therefore, the results should not be interpreted as evidence of diagnostic-deployment readiness or a replacement of clinician interpretation.

This study provides a controlled transportability benchmark for engineered ECG features with four main contributions. First, it reports a cardiologist-annotated hospital dataset of 1749 usable ECG recordings, including explicit accounting of broken-data exclusions, class imbalance, repeated recordings, and two-cardiologist label review. Second, it defines a matched healthy-versus-MI task in PTB-XL using 14,982 recordings under the official development, validation, and test folds. Third, it compares 11 engineered feature families and 5 classifier families under a common preprocessing, imputation, scaling, balancing, hyperparameter-search, and threshold-selection framework. Fourth, it separates strict source-only cross-dataset transfer from a secondary target-threshold selection analysis, so that model transportability and operating-threshold transportability are not conflated.

## 2. Materials and Methods

This study was designed as a binary healthy-versus-MI ECG classification and transportability analysis using an original cardiologist-annotated hospital dataset and the public PTB-XL dataset. The primary analyses consisted of the two internal evaluations and the two strict source-only cross-dataset transfer evaluations. In the strict source-only transfer setting, all preprocessing, feature extraction, model fitting, hyperparameter selection, and threshold selection were completed using the source dataset only. Several related ECG-MI studies have incorporated target-domain retraining, fine-tuning, architecture transfer, or digitization-specific retraining [23,24,25]. The present study intentionally left these adaptation steps out of the main analysis so that model transportability and adaptation to the target setting are not mixed together. This design was chosen to isolate source-only transportability and to investigate whether fixed engineered feature families and classical models retain their performance when transferred between independently labeled clinical and public ECG datasets. A secondary target-threshold analysis changed only the operating threshold using target labels; it did not refit the probability model and was reported separately from strict source-only transfer [27,28]. The methods are reported to align with current expectations for prediction-model and diagnostic-AI studies, including transparent data description, separation of development, validation, and testing, explicit handling of applicability, and limited claims about clinical use [29,30,31,32].

Throughout the paper, external validation refers to evaluation in a separate data source or setting. Source-only transfer refers to external testing after all model and threshold choices have been made with source-dataset labels only. Target-threshold selection refers only to re-selecting the operating threshold using target labels while keeping the fitted probability model fixed. Transfer learning refers to retraining, fine-tuning, architecture transfer, or other target-domain adaptation and is not part of the strict source-only analysis.

### 2.1. Data Sources

#### 2.1.1. Cardiologist-Annotated Hospital Dataset

The original hospital dataset was collected from patients who presented to the hospital with chest pain and contained 1955 indexed ECG records from 1579 patients. The recordings were acquired in 2024 using Nihon Kohden ECG devices (Nihon Kohden Corporation, Tokyo, Japan) and were stored as 10 s, 12-lead ECG recordings with a sampling frequency of 500 Hz. The recordings were exported in XML/HL7 aECG format. The records were accepted only when all 12 leads and 5000 samples per lead were present. Two cardiologists labeled the records using an originally developed Python-based review interface that displayed the 12-lead ECG and stored the assigned class labels. Discordant or uncertain cases were revisited during the labeling process before the final dataset was created. The records with short or incomplete samples, zero or near-zero amplitude, non-recoverable missing leads, and severe artifact were labeled as broken data. These broken-data records (n=206) were excluded before the binary analysis since they prevented ECG interpretation. The final hospital dataset contained 1749 usable recordings from 1434 patients, including 1550 Healthy recordings and 199 MI recordings, corresponding to an MI prevalence of 11.38%. Female and male record counts were 666 and 1083, respectively. Before binary merging, the MI labels consisted of anterior MI (n=131), inferior MI (n=49), and lateral MI (n=19) and these classes were collapsed into a single MI class for compatibility with the PTB-XL binary task. Repeated recordings were present, so all hospital splits were grouped by patient.

Explicit inclusion criteria for the hospital source were: presentation within the chest-pain ECG workflow, acquisition in 2024 on the available 12-lead hospital ECG system, availability of a 10 s 500 Hz XML/HL7 aECG record, interpretable waveforms in the canonical 12 leads, and cardiologist assignment to Healthy or MI for the binary task. Exclusion criteria were: broken-data status, incomplete waveform length, missing or unrecoverable leads, zero or near-zero amplitude, severe artifact preventing interpretation, or absence of a usable binary Healthy/MI label after cardiologist review. Other non-MI pathological ECG categories were not modeled in this binary analysis.

**Figure 1:**
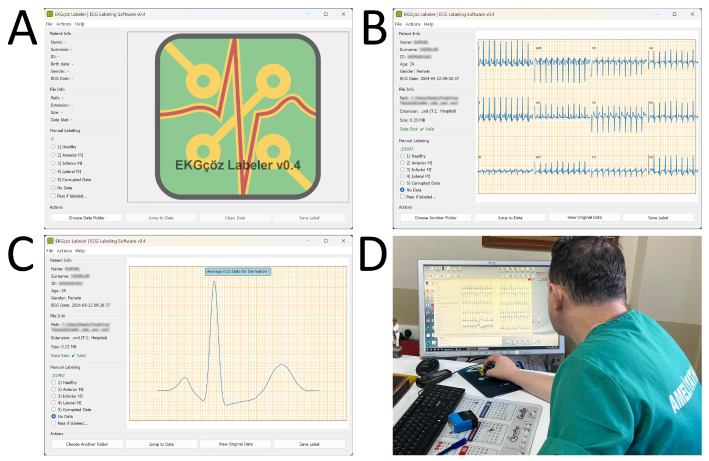
Custom ECG labeling software used in the hospital dataset workflow. Panel (**A**) shows the idle interface before an ECG record is loaded. Panel (**B**) shows the 12-lead ECG review screen used for record-level inspection. Panel (**C**) shows the averaged single-heartbeat view when clicked on a lead. Panel (**D**) shows a cardiologist using the software during the labeling process.

The hospital labels were prepared through a dedicated graphical user interface application to collect cardiologist reviews. The application was developed to display the 12-lead ECG, allow case navigation, support class assignment, and preserve saved outputs for the dataset build. In the same project line, a separate decision-support application was prepared to load a trained model and return a predicted class together with model confidence for clinical review. That application was designed as a support tool for cardiologists, not as an autonomous diagnostic system. The labeling interface and review workflow are shown in Figure 1.

#### 2.1.2. PTB-XL Binary Subset

PTB-XL is a widely used public 12-lead ECG dataset for ECG signal-processing studies because of its large sample size, structured diagnostic annotations, standardized metadata, and recommended folds for model development and testing. The dataset includes 21,837 records from 18,885 patients [34]. In the matched binary subset used in this study, female and male record counts were close with 7793 and 7189, respectively, and are reported at the record level. The official split was preserved, with folds 1–8 for training, fold 9 for validation, and fold 10 for testing [35].

For PTB-XL, the Healthy class corresponded to the selected control class, and MI was defined from the PTB-XL MI-superclass SCP statements listed in Table 1. The binary labels were derived from the SCP Codes field in the dataset metadata. The matched binary subset in this study retained 14,982 recordings from 13,436 patients: 9513 Healthy recordings and 5469 MI recordings, corresponding to an MI prevalence of 36.50%. A record was assigned to the MI class if it contained at least one SCP statement mapped to the PTB-XL diagnostic superclass MI: AMI, ALMI, ASMI, ILMI, IMI, INJAL, INJAS, INJIL, INJIN, INJLA, IPLMI, IPMI, LMI, or PMI. Most of these statements explicitly describe myocardial infarction location. The INJ statements describe subendocardial injury, but they were also included because their PTB-XL diagnostic superclass is MI. All the SCP statements with their number of records and descriptions are shown in Table 1. A record was assigned to the Healthy class only when NORM was present and none of the MI-superclass codes was present. Records containing neither NORM nor an MI-superclass code were excluded from the binary subset.

This PTB-XL label construction is not identical to the hospital clinical label construct. PTB-XL MI-superclass statements may include old infarction, location statements, and injury-related codes, whereas the hospital dataset was assembled from a chest-pain clinical workflow with cardiologist review. Therefore, differences in label definition, disease stage, and clinical context were treated as plausible contributors to the transfer gap rather than as noise-free measurements of the same endpoint. From the original 21,837 PTB-XL records, 6855 records were excluded by the selected binary mapping because they contained neither the retained Healthy definition nor any selected MI-superclass SCP code; these excluded records do not have a Healthy/MI class distribution under the present binary rule.

Table 2 summarizes the final record-level characteristics of the hospital dataset and the matched PTB-XL binary subset used in the main analysis. The differences between these datasets, such as patient count and MI proportion, were retained in the analysis because the aim was to evaluate transportability across independently labeled clinical and public ECG sources.

Because the hospital source came mainly from one institution and one ECG acquisition system, the transfer gap was interpreted as the combined effect of at least three nonexclusive components: label/prevalence differences, acquisition and device-related feature differences, and true ECG feature-distribution shift. Aggregate raw-feature summaries such as ST-amplitude distributions, fiducial duration summaries, and QRS-related interval summaries could be reported without releasing private ECG records. In the revised Supplementary Material, the available aggregate quality summaries document feature-family missingness and ST-extraction fallback behavior, helping separate data-quality effects from broader label, acquisition, and feature-shift explanations.

**Figure 2:**
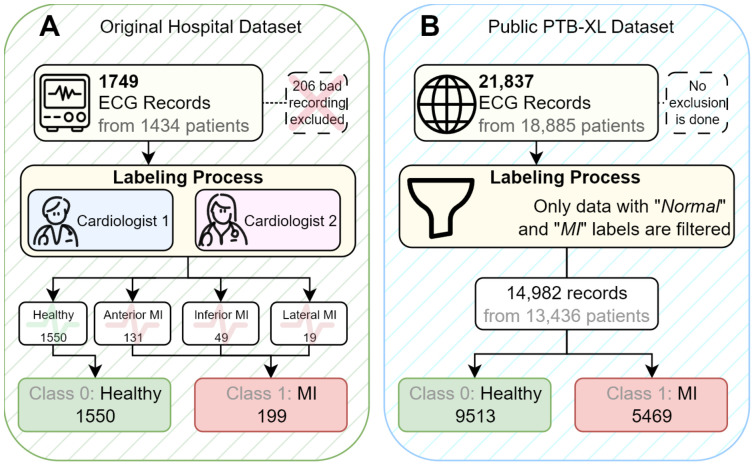
Construction of the two binary healthy-versus-MI datasets used in the study. Panel (**A**) summarizes the hospital-data workflow and Panel (**B**) summarizes the PTB-XL workflow from the original PTB-XL release to the matched binary subset used in this study.

The dataset-construction pathways for the hospital and PTB-XL analyses are summarized in Figure 2.

### 2.2. Signal Preprocessing and Feature Extraction

Before feature extraction, all ECGs were reordered to the canonical sequence I, II, III, aVL, aVR, aVF, and V1 to V6. For signal processing, 500 Hz ECGs were used and no additional resampling was performed. Hospital XML/HL7 aECG records were required to contain 12 leads with 5000 samples per lead, and PTB-XL records were loaded from the high-resolution WaveForm Database (WFDB) filenames. Records with missing canonical leads or incompatible hospital waveform length were excluded. Each lead was filtered using zero-phase forward-backward filtering with a third-order Butterworth bandpass from 0.5 to 40 Hz, and then median-centered. Hospital XML waveforms were stored as digital sample values with origin 0 μV and scale 1.25 μV per digital count. During waveform loading, these digital values were converted to physical millivolt units using ${\mathrm{sample}}_{\mathrm{mV}}=\mathrm{digit}\times 0.00125$ before preprocessing and feature extraction. PTB-XL WFDB records were likewise read as physical mV signals, so amplitude-preserving features in both datasets were derived from signals expressed in mV. For the normalized feature branch, each lead was divided by its own standard deviation. After feature extraction, missing feature values in the model-input matrix were filled using medians estimated from the training data, and missingness indicators were added to record which values had originally been unavailable. Scaling was applied only for logistic regression and support vector machine (SVM). These preprocessing steps were fitted on the training data and then applied unchanged to the validation, internal-test, and target-test data.

A project-specific custom ST-segment detector was used as one of the ST extraction branches [37]. ST-segment features were summarized per lead as the number of valid segments and the mean and standard deviation of ST amplitude, duration, slope, and trapezoidal area after baseline subtraction. The baseline was the median signal from 80 to 20 samples before the reference R peak. The custom strict detector used shared R peaks, estimated the S point as the minimum in the R to R + 70 sample window, and selected a T-related endpoint in the S + 40 to S + 180 sample window using the strongest gradient, with an amplitude-based fallback. The NeuroKit2 branch used NeuroKit ECG cleaning and discrete wavelet transform (DWT) delineation to pair S peaks with T onsets [37,38]. The shared fallback used the same baseline, estimated an approximate J point 10 samples after the detected S point, searched a T endpoint from J + 20 to J + 140 samples, and summarized the baseline-subtracted J to T segment. The fallback policy was used to avoid empty ST-feature entries and the quality-control (QC) counts were interpreted as measures of extraction completeness. The number of fallback-recovered segments was also analyzed to compare differences between the ST extraction methods. The engineered feature families used in the model comparison are summarized in Table 3.

The number of extracted features before missing-value handling was 96 for beat-template morphology, 96 for repolarization descriptors, 63 for lead-group spatial descriptors, 96 for fiducial interval descriptors, 96 for custom ST descriptors, 96 for NeuroKit ST descriptors, 192 for the dual ST descriptor bank, 255 for the compact clinical feature bank, 495 for the broad hybrid feature bank, 591 for the broad hybrid + ST bank, and 26 for lead-connectivity descriptors.

### 2.3. Classifiers Used and Hyperparameter Search

Five classifier families were compared to cover both simple linear baselines and nonlinear tree-based models: logistic regression, radial-basis SVM, random forest, XGBoost, and Extra Trees. Logistic regression and SVM used scaled inputs, whereas the tree-based models used the imputed feature values without scaling. Hyperparameter tuning was performed only on the validation split defined by each protocol. The search space was intentionally kept small because the aim was to compare feature-family transportability rather than to maximize one model through extensive optimization. For logistic regression, the strength of regularization was varied from stronger to weaker regularization. For SVM, the main penalty strength was varied and the kernel width was allowed to follow standard data-dependent settings. For the tree-based models, a fixed small random-search budget varied common settings such as tree depth, number of trees, learning rate for boosting, subsampling, minimum leaf size, and feature sampling.

In architectural terms, logistic regression was used as an L2-regularized linear probabilistic classifier; the SVM used a radial-basis-function kernel; random forest and Extra Trees were bagged decision-tree ensembles differing in the degree of split randomization; and XGBoost was a gradient-boosted decision-tree ensemble. The parameters considered for the linear and kernel models included regularization strength and, for SVM, kernel-width handling. The parameters considered for tree ensembles included the number of estimators, maximum depth, minimum samples or child weight, feature subsampling, and, for XGBoost, learning rate and row/column subsampling. Exact candidate grids and search budgets are reported in Supplementary Table S3.

### 2.4. Imbalance Handling and Hospital Policy Selection

The hospital dataset label distribution was imbalanced with 199 MI and 1550 Healthy recordings in the final binary dataset. Imbalance handling was selected in a preliminary experiment before the main feature-model analyses. Four policies were compared: no balancing, class-weight handling, mild random oversampling, and moderate random oversampling. The selected hospital policy was mild random oversampling, which increased the minority class during training to approximately one MI sample for every three Healthy samples. Oversampling was applied only after the train/validation or train/test split and only to the training data; it was never applied to validation, internal-test, or target-test data. The PTB-XL-trained analyses used sample weighting only. The current analysis code uses one balancing mechanism per model fit, which avoids mixing class weights and oversampling in the same final training call. Table 4 shows that the mean balanced-accuracy gain over no balancing was small, but the selected policy improved sensitivity in the low-prevalence hospital setting. Because the balanced-accuracy differences between imbalance policies were small, the selected mild-oversampling policy should be interpreted as a pragmatic source-training choice rather than as a robustly superior clinical preprocessing rule.

### 2.5. Evaluation Protocols, Metrics, and Statistical Analysis

The evaluation included internal analyses within each dataset and strict source-only cross-dataset transfer analyses between datasets. For the hospital internal analysis, 5-fold stratified group splitting was performed at the patient level to avoid placing recordings from the same patient in both training and test sets. In each fold, the training portion was further divided into a grouped training set and a grouped validation set, and the validation set was used for hyperparameter and threshold selection. For the PTB-XL internal analysis, the official benchmark split was used. Folds 1–8 were used for training, fold 9 for validation, and fold 10 for testing.

The primary cross-dataset analyses evaluated strict source-only transfer in both directions. In the hospital-to-PTB-XL direction, 1399 hospital recordings were used for source training, 350 hospital recordings were used for source validation, and 1513 PTB-XL fold-10 recordings were used only for target testing. In the PTB-XL-to-hospital direction, 11,974 PTB-XL recordings were used for source training, 1495 PTB-XL recordings were used for source validation, and 1399 hospital recordings were used only for target testing. In the strict source-only transfer analyses, all model fitting, hyperparameter selection, and operating-threshold selection were completed using only the source dataset. The target dataset was used once, after source-side selection was complete, for final evaluation only.

A secondary target-threshold analysis was also performed. In this analysis, the trained source model and its probability outputs were kept fixed, but the operating threshold was re-selected using target labels. This analysis was reported separately because it uses target-label information. Re-selecting the threshold can change threshold-dependent metrics such as balanced accuracy, sensitivity, specificity, precision, and F1 score. However, it cannot change threshold-free metrics such as receiver operating characteristic area under the curve (ROC-AUC) and precision–recall area under the curve (PR-AUC), or the Brier score, because the underlying predicted probabilities remain unchanged.

**Figure 3:**
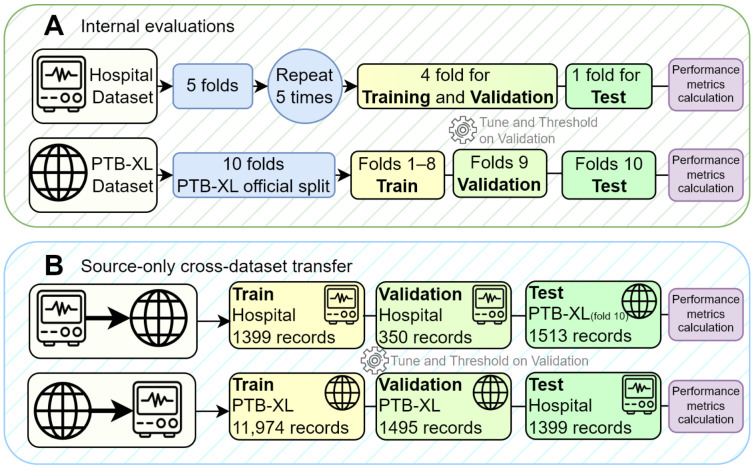
Overview of the study design. Panel (**A**) shows the internal evaluation workflows for the hospital dataset and PTB-XL. Panel (**B**) shows the strict source-only cross-dataset transfer workflows in both directions, with source-side training and validation followed by one-time target-side testing.

The overall evaluation design, including the internal analyses and the strict source-only cross-dataset transfer analyses, is summarized in Figure 3.

Evaluation combined quantitative point metrics with curve-based qualitative diagnostics. The quantitative component used operating-point metrics at the selected threshold, threshold-free ranking metrics, probability-error summaries, and bootstrap uncertainty. The qualitative component used ROC and precision–recall curves to inspect separation, curve shape, and evidence of score clustering or threshold mismatch across transfer directions.

The main operating-point metric was balanced accuracy (BA) because the hospital dataset was class-imbalanced, with many more Healthy recordings than MI recordings. Sensitivity and specificity were reported alongside BA to show whether a model favored MI detection or Healthy classification at the selected operating threshold. ROC-AUC and PR-AUC were used as threshold-free ranking metrics. PR-AUC was included because it is sensitive to class prevalence and is informative when the positive class is uncommon. The Brier score was used to summarize probability error, and confusion counts were reported for the leading results.

For a threshold τ, predicted MI labels were defined as ${\hat{y}}_{i}\left(\tau \right)=1$ when $p_{i}\geq \tau$ and ${\hat{y}}_{i}\left(\tau \right)=0$; otherwise, where $p_{i}$ is the predicted probability of MI for record *i*. The confusion-matrix counts were defined with MI as the positive class: TP and FN refer to correctly and incorrectly classified MI recordings, whereas TN and FP refer to correctly and incorrectly classified Healthy recordings. Sensitivity, specificity, and balanced accuracy were then calculated using Equations (1)–(3).

(1) $$\mathrm{Sensitivity}\left(\tau \right)=\frac{\mathrm{TP}(\tau )}{\mathrm{TP}(\tau )+\mathrm{FN}(\tau )},$$

(2) $$\mathrm{Specificity}\left(\tau \right)=\frac{\mathrm{TN}(\tau )}{\mathrm{TN}(\tau )+\mathrm{FP}(\tau )},$$

(3) $$\mathrm{BA}\left(\tau \right)=\frac{\mathrm{Sensitivity}(\tau )+\mathrm{Specificity}(\tau )}{2}.$$

The operating threshold was selected on the source-validation split by maximizing validation BA over a fixed grid of 37 thresholds from 0.05 to 0.95, as shown in Equation (4).

(4) $${\hat{\tau }}_{\mathrm{source}}=\arg\max_{\tau \in T}{\mathrm{BA}}_{\mathrm{val}}\left(\tau \right).$$

The same source-selected threshold was then applied unchanged during internal testing and strict source-only target testing. In the secondary target-threshold analysis, only the threshold-selection step in Equation (4) was repeated using target labels; model parameters and predicted probabilities were not changed. The Brier score was calculated using Equation (5):

(5) $$\mathrm{Brier}=\frac{1}{N}\sum_{i=1}^{N}{\left(p_{i}-y_{i}\right)}^{2},$$

where $y_{i}\in \left\{0,1\right\}$ is the reference label for record *i*. Lower Brier scores indicate smaller probability error. Point estimates for the leading results were accompanied by percentile bootstrap confidence intervals. Patient-level bootstrap sampling was used when patient identifiers were available; otherwise, record-level resampling was used.

All analyses were performed in Python 3.13.12. Numerical processing and data handling used NumPy 2.4.3, pandas 2.3.3, and SciPy 1.17.1. Machine learning models were implemented with scikit-learn 1.8.0 and XGBoost 3.2.0, and ECG signal-processing functions used NeuroKit2 0.2.13. Figures were generated with Matplotlib 3.10.8.

## 3. Results

The results are presented in four steps. First, internal performance within each dataset is summarized to show the apparent performance ceiling under matched source conditions. Second, strict source-only transfer is evaluated in both directions. Third, a secondary target-threshold analysis separates score ranking from threshold calibration. Fourth, feature-extraction reliability and software workflow components are summarized.

### 3.1. Within-Dataset Classification Performance

The best hospital result was obtained by the fiducial interval descriptor family with Extra Trees. This model reached a balanced accuracy of 0.775, ROC-AUC 0.855, PR-AUC 0.479, sensitivity 0.724, and specificity 0.827. The selected threshold was 0.205. The confusion counts were 1282 true negatives, 268 false positives, 55 false negatives, and 144 true positives. The bootstrap mean balanced accuracy was 0.774 with 95% confidence interval (CI) 0.734–0.810. The next two hospital results were the dual ST descriptor family with Extra Trees (balanced accuracy 0.763) and the broad hybrid + ST bank with Extra Trees (balanced accuracy 0.762).

**Figure 4:**
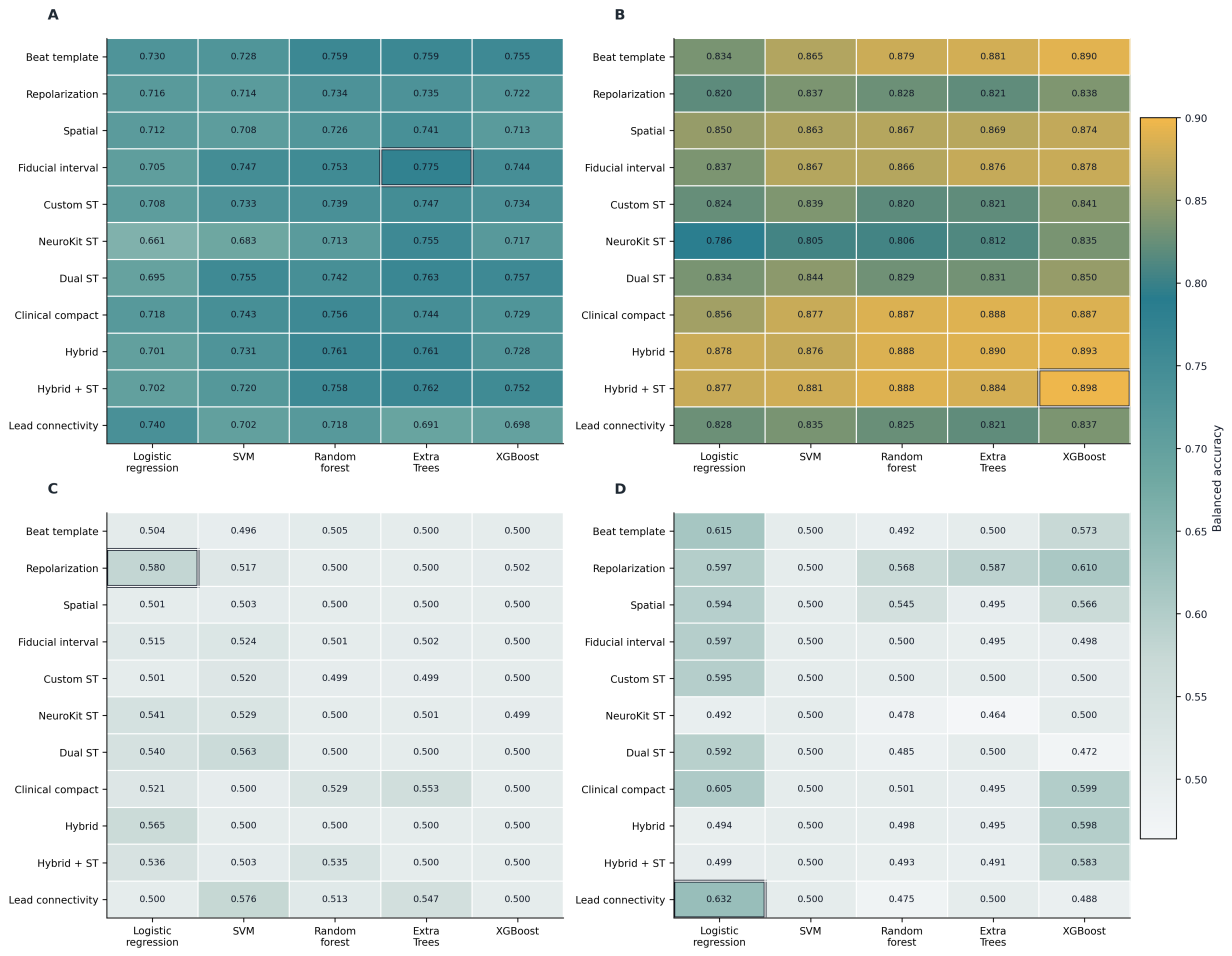
Balanced-accuracy heatmaps for the main experiments. Panel (**A**) shows within-hospital performance across feature families and model families. Panel (**B**) shows within-PTB-XL performance using the same feature–model comparison. Panel (**C**) shows strict source-only cross-dataset transfer from the hospital dataset to PTB-XL. Panel (**D**) shows strict source-only cross-dataset transfer from PTB-XL to the hospital dataset. A common color scale is used across all panels to make the drop from internal evaluation to external transfer directly comparable. The full numeric balanced-accuracy values are also provided in the Supplementary Material.

**Figure 5:**
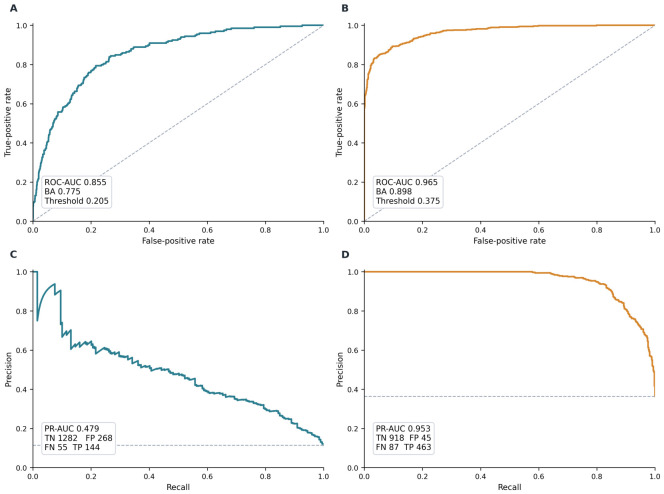
ROC and precision–recall curves for the best-performing internal models. Panels (**A**,**C**) show the hospital dataset results, and Panels (**B**,**D**) show the PTB-XL dataset results. AUC values and confusion-count summaries are reported within the panels. MI is the positive class and Healthy is the negative class; therefore, TP and FN refer to MI recordings, whereas TN and FP refer to Healthy recordings.

The best PTB-XL result was obtained by the broad hybrid + ST bank with XGBoost. This model reached a balanced accuracy of 0.898, ROC-AUC of 0.965, PR-AUC of 0.953, sensitivity of 0.842, and specificity of 0.953. The selected threshold was 0.375. The confusion counts were 918 true negatives, 45 false positives, 87 false negatives, and 463 true positives. The bootstrap mean balanced accuracy was 0.898 with 95% CI 0.877–0.916. The next two PTB-XL results were the broad hybrid bank with XGBoost (balanced accuracy 0.893) and beat-template morphology with XGBoost (balanced accuracy 0.890). Table 5 summarizes the top internal rows, and Figure 4 and Figure 5 show the corresponding feature–model heatmaps and internal ranking curves.

Clinically, the leading hospital interval family reflects timing and duration information from the representative beat, whereas the leading PTB-XL hybrid + ST family combines morphology, interval, repolarization, spatial, and ST-segment summaries. This difference supports the interpretation that successful engineered features may depend on the dataset label construct and acquisition setting rather than on one universally dominant feature family.

### 3.2. Strict Source-Only Cross-Dataset Transfer

The strict source-only cross-dataset transfer results were much lower than the internal results. When the training and validation data came from the hospital dataset and the test data came from PTB-XL, the best result was produced by repolarization descriptors with logistic regression. This model reached a balanced accuracy of 0.580, ROC-AUC of 0.774, PR-AUC of 0.726, sensitivity of 0.164, and specificity of 0.996 at a threshold of 0.15. The confusion counts were 959 true negatives, 4 false positives, 460 false negatives, and 90 true positives. The bootstrap mean balanced accuracy was 0.580 with 95% CI 0.563–0.599. The low false-positive count is attractive, but the sensitivity drop shows that the hospital-trained operating point did not transfer well to PTB-XL. The difference between the ROC-AUC of 0.774 and sensitivity of 0.164 at the source-selected threshold indicates that the problem was not only feature informativeness but also operating-threshold transportability. This sensitivity would not be acceptable for clinical MI screening or decision support if used without recalibration, target-setting validation, and prospective clinical evaluation.

When the training and validation data came from PTB-XL and the test data came from the hospital dataset, the best result was produced by lead-connectivity descriptors with logistic regression. This model reached a balanced accuracy of 0.632, ROC-AUC of 0.635, PR-AUC of 0.161, sensitivity of 0.553, and specificity of 0.710 at a threshold of 0.45. The confusion counts were 880 true negatives, 360 false positives, 71 false negatives, and 88 true positives. The bootstrap mean balanced accuracy was 0.632 with 95% CI 0.588–0.673. The reverse direction therefore stayed more balanced in sensitivity and specificity, but the absolute performance was still well below the internal PTB-XL result.

The strongest alternatives remained close but did not change the interpretation. For hospital to PTB-XL, the next two models were lead-connectivity descriptors with SVM (balanced accuracy 0.576) and the broad hybrid feature bank with logistic regression (balanced accuracy 0.565). For PTB-XL to hospital, the next two models were beat-template morphology with logistic regression (balanced accuracy 0.615) and repolarization descriptors with XGBoost (balanced accuracy 0.610). Table 6 summarizes the candidate contributors to directional transfer asymmetry and Table 7 summarizes the leading strict source-only transfer rows.

Most feature-family blocks had no missing values; the largest missingness fraction was observed for lead-connectivity features, with 0.044% missingness in the hospital dataset and 0.0013% in PTB-XL. ST-feature extraction also produced complete final outputs after fallback recovery, with fallback required for 440 of the 20,988 hospital NeuroKit lead slots (2.10%) and 3393 of the 179,784 PTB-XL NeuroKit lead slots (1.89%). These values make a pure missingness explanation unlikely, while leaving label-construct, acquisition, prevalence, feature-distribution, and threshold-calibration differences as plausible contributors to the asymmetric transfer results.

**Figure 6:**
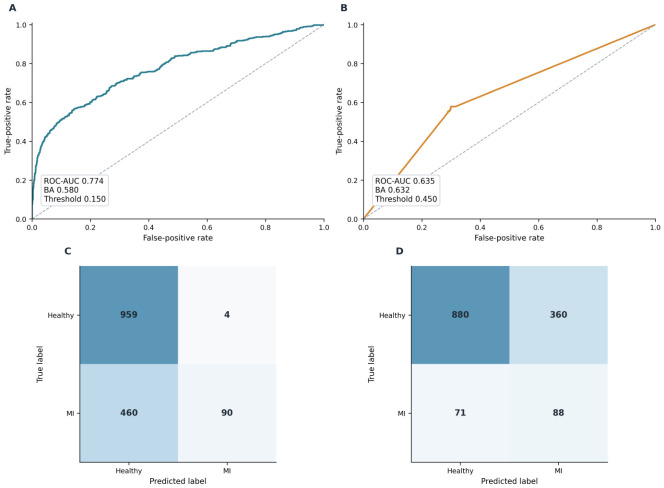
Strict source-only cross-dataset transfer performance for the two transfer directions. Panel (**A**) shows the ROC curve for hospital-to-PTB-XL transfer, and Panel (**B**) shows the ROC curve for PTB-XL-to-hospital transfer. Panel (**C**) shows the confusion matrix for hospital-to-PTB-XL transfer, and Panel (**D**) shows the confusion matrix for PTB-XL-to-hospital transfer. The piecewise-linear appearance of the PTB-XL-to-hospital ROC curve is consistent with clustered or coarsely separated predicted scores, as discussed in the Results.

Figure 6 summarizes the strict source-only transfer behavior in both directions. In hospital-to-PTB-XL transfer, the ROC curve showed that the source-trained model retained some ranking ability on the target dataset, although the source-selected operating threshold produced low sensitivity. In PTB-XL-to-hospital transfer, the ROC curve had a piecewise-linear appearance, suggesting that the transported model assigned clustered or coarsely separated prediction scores to the hospital records rather than a finely graded score distribution. These patterns support the interpretation that cross-dataset transportability should be assessed not only by ranking metrics such as ROC-AUC, but also by score behavior and operating-threshold performance.

### 3.3. Secondary Target-Threshold Selection

The secondary target-threshold analysis changed the selected threshold in 104 of 110 aligned model rows, and the threshold-tuned balanced accuracy changed in 76 rows. As expected, ROC-AUC, PR-AUC, and Brier score did not change when only the threshold changed. The best threshold-selected hospital-to-PTB-XL result remained the repolarization descriptor family with logistic regression. Balanced accuracy increased from 0.580 to 0.682, a gain of 0.102, and the selected threshold moved to 0.05. Under this threshold, sensitivity increased to 0.405 while specificity remained 0.958. For PTB-XL to hospital, the best threshold-selected result became the broad hybrid + ST bank with XGBoost. Balanced accuracy increased from 0.632 to 0.640, a gain of 0.009, with selected threshold being 0.925, sensitivity 0.535, and specificity 0.746. Table 8 separates these secondary threshold-selected values from the strict source-only rows.

These results show that part of the transfer problem was an operating-threshold mismatch. However, the analysis used target labels for threshold selection and therefore remains secondary. It should not be read as a strict source-only transfer result.

Threshold calibration should therefore be viewed as a distinct validation requirement beyond ROC-AUC. A model can preserve partial ranking ability in the target data while its source-selected operating threshold produces unacceptable sensitivity or specificity. External validation of ECG-MI models should report both threshold-free ranking metrics and clinically relevant operating-point metrics under clearly specified threshold-selection rules.

### 3.4. Feature-Extraction Reliability for ST Detection

The ST quality-control analysis was used to verify that the selected feature-extraction policy produced complete lead-level ST outputs before model comparison. The custom ST extractor produced complete outputs in both datasets without requiring fallback correction, with 20,988 complete hospital lead slots and 179,784 complete PTB-XL lead slots. In contrast, strict NeuroKit-based delineation produced 20,548 complete hospital lead slots and 176,391 complete PTB-XL lead slots, leaving 440 and 3393 lead slots to be recovered by the shared fallback rule, respectively. These fallback-recovered slots corresponded to 2.10% of hospital NeuroKit lead slots and 1.89% of PTB-XL NeuroKit lead slots. After fallback, no empty ST slots remained in the selected common-fallback version.

### 3.5. Decision-Support Software Components

The labeling interface and the decision-support application were not part of the main classifier-ranking analysis, but they are relevant to the broader project workflow. The labeling interface supported cardiologist review during the hospital dataset build, and the decision-support application was prepared to display model outputs in a review setting. The present paper does not make claims of clinical deployment. Instead, it documents the classification study that informed those translational tools.

## 4. Discussion

The discussion interprets the transfer gap as a methodological and clinical-scope finding. It separates internal performance from external source-only performance, distinguishes engineered-feature benchmarking from deep-learning transfer studies, and clarifies why the observed operating points are not sufficient for clinical decision support.

### 4.1. Main Findings

The primary finding was a substantial divergence between internal validation performance and source-only transportability. The best internal balanced accuracy was 0.775 in the cardiologist-annotated hospital dataset and 0.898 in PTB-XL, whereas the best strict source-only cross-dataset balanced accuracy was 0.580 for hospital-to-PTB-XL transfer and 0.632 for PTB-XL-to-hospital transfer. This pattern should be interpreted as evidence of dataset-shift sensitivity in engineered ECG-MI models, not as evidence that the internal classifiers were weak. The study therefore contributes a transparent source-only transportability benchmark and shows why internal validation, target-threshold selection, and target-adapted transfer should be reported separately.

A second important result is that the leading feature family changed with the evaluation setting. Fiducial interval descriptors were best in the hospital dataset. A broad hybrid feature bank with ST information was best in PTB-XL. Repolarization descriptors were best for hospital-to-PTB-XL transfer, while lead-connectivity descriptors were best for PTB-XL-to-hospital transfer. Because the top-performing configurations changed across settings, the study does not support a universal claim that one handcrafted feature family is dominant for all MI ECG classification problems.

### 4.2. Relation to Published ECG MI Studies

Several recent ECG-AI studies have reported high performance estimates, especially for focused STEMI or occlusion MI tasks. Zhao et al. reported external AUC 0.9954 for AI-based STEMI detection [5]. Lee et al. reported 92.1% accuracy with 95.4% sensitivity and 91.8% specificity in a clinically validated STEMI model [6]. Al-Zaiti et al. showed strong multi-site performance for occlusion MI risk stratification [7]. These studies are important reference points, but they address different tasks, datasets, label definitions, or care pathways.

The three most similar papers also clarify how the present study should be positioned. EvoMBN is methodologically relevant because it uses 12-lead ECGs, inter-patient evaluation, and PTB-to-PTB-XL architecture transfer; however, it is a deep multi-branch architecture-search study rather than an engineered-feature transportability study [23]. The paper-ECG digitization study by Rai et al. is relevant from a translational perspective because it combines PTB-XL, ECG-image data, and hospital paper ECG data; however, its main improvement comes from digitization plus training-retraining on target-style data [24]. The lightweight pathology-guided study by Bao et al. is close in its use of PTB-XL, lead selection, inter-patient evaluation, and a clinical dataset; however, the clinical transfer stage uses target-domain fine-tuning rather than strict source-only testing [25]. Table 9 summarizes these distinctions.

From this perspective, the modest strict-transfer values are a central transportability finding rather than a secondary weakness. They quantify the practical gap between strong internal validation, target-adapted transfer learning, and strict source-only transfer across datasets. This is the type of issue emphasized by external-validation and diagnostic-AI reporting guidance [27,28,29,30,31]. The results are also consistent with broader evidence that dataset shift can degrade machine learning biomarker performance even when the signal source appears similar [33]. The present results should therefore be read as complementary to deep-learning studies: they do not claim to outperform optimized neural models, but they quantify transportability for transparent feature families under a deliberately stricter transfer rule.

### 4.3. Feature-Family Variation Across Validation Settings

The changes in leading feature family should not be interpreted as causal evidence or as evidence that any feature family is globally superior. Rather, they suggest that MI-related information was distributed across multiple components of the ECG representation. This interpretation is plausible because MI-related ECG behavior spans ST-segment changes, T-wave and repolarization abnormalities, interval changes, morphology, and multi-lead relationships [1,3,4,17,20]. The hospital dataset favored interval descriptors and tree models, while PTB-XL favored a broader hybrid bank with ST information and boosting. The top-performing transfer configurations differed again. This pattern suggests that feature diversity may be more informative than reliance on a single predefined feature family.

The clinical meaning of the most successful families is consistent with this interpretation. Fiducial intervals summarize timing of depolarization and repolarization-related landmarks, ST descriptors target ischemia-relevant segment behavior, repolarization descriptors capture T-wave morphology and polarity, and lead-connectivity descriptors summarize cross-lead coherence. Their variable success across settings suggests that dataset-specific label and acquisition factors can change which clinically interpretable ECG component is most predictive.

The hospital-to-PTB-XL result also illustrates why balanced accuracy and confusion counts should be reported together. The repolarization-plus-logistic-regression model had strong specificity (0.996) and acceptable ROC-AUC (0.774), yet its sensitivity was only 0.164 at the selected source-derived threshold. The reverse transfer was more balanced, but still remained below the internal PTB-XL result. These findings indicate that ranking performance and operating-threshold behavior should be interpreted jointly when the dataset changes.

### 4.4. Interpretation of the Secondary Target-Threshold Analysis

The secondary target-threshold analysis showed that part of the transfer problem was an operating-threshold mismatch. This was most visible for hospital-to-PTB-XL transfer, where balanced accuracy increased from 0.580 to 0.682 after threshold re-selection on target labels. However, the probability model itself did not change, so the ranking metrics were fixed. This distinction is methodologically important. A threshold-tuned transfer result may be informative as a secondary analysis, but it should not be interpreted as equivalent evidence to strict source-only transfer [27,28].

### 4.5. Strengths, Limitations, and Translational Value

The study has several strengths. First, it includes a cardiologist-annotated hospital dataset rather than relying only on a public benchmark. Second, it keeps the preprocessing, feature-family comparison, threshold policy, and model search structure matched across experiments. Third, it uses patient-grouped hospital splitting and explicit source-only transfer as the main transportability design. Fourth, it reports quality-control accounting for the ST blocks instead of treating feature extraction as a black box.

Several limitations should be considered. The private data came from one hospital source, so the paper does not solve multi-site generalization. The binary healthy-versus-MI design excluded other non-MI pathological ECGs and should not be interpreted as an all-comers diagnostic or emergency-triage task. MI subtypes were merged into one MI class for the main binary task, and hospital labels and PTB-XL SCP statements were harmonized only at the binary level. Demographic fields were not modeled because the main design was ECG-only. Age was retained only as descriptive metadata and was not used for model training. Hospital XML amplitudes were amplitude-scaled to mV during waveform loading, so the reported transportability gap should not be attributed to an unhandled raw-count versus physical-voltage unit mismatch. Nevertheless, amplitude scaling does not remove other single-site hospital limitations, acquisition differences, label-source differences, feature-distribution shifts, or threshold-transportability problems. The leading transfer rows were selected from a prespecified benchmark grid, so they should be interpreted as highest observed benchmark rows, not as external validation of one locked final model. No deep-learning baseline was included in the matched experiment grid, so the paper should not be framed as a full classical-versus-deep comparison. This point is important because the most similar neural studies use architecture transfer, target-domain retraining, or fine-tuning, which answers a different question from strict source-only transfer [23,24,25]. Finally, the decision-support application described here is a project output, not evidence of clinical deployment.

Despite these limitations, the software components provide practical value within the project workflow. The cardiologist labeling interface supported creation of the hospital dataset under expert review, and the model-loading application illustrates a possible review-support workflow. However, prospective multi-site testing, human-factors/interface validation, and regulatory review would be required before any clinical-use claim can be made [39]. A supplementary comparison table separating strict source-only transfer, secondary target-threshold analysis, target-domain retraining, and transfer learning may also help prevent these validation modes from being conflated in the ECG-MI literature.

## 5. Conclusions

This two-dataset ECG study provides a strict source-only transportability benchmark for engineered 12-lead ECG features in healthy-versus-MI classification. Internal balanced accuracy reached 0.775 in the cardiologist-annotated hospital dataset and 0.898 in PTB-XL, but strict source-only cross-dataset performance was substantially lower, with highest observed balanced accuracy values of 0.580 for hospital-to-PTB-XL transfer and 0.632 for PTB-XL-to-hospital transfer without target-domain retraining. The leading feature family also changed across datasets and transfer directions, indicating that no single engineered representation was universally dominant. The main contribution is therefore a transparent measurement of how internal performance, feature-family rankings, ranking metrics, and operating thresholds change when ECG-MI models are transported between clinical and public datasets, including the asymmetric behavior observed between the two transfer directions. Prospective multi-site validation is required before clinical-use claims can be made. These results should be read as highest observed benchmark performance across a prespecified feature-family/model grid, not as performance of a locked final model ready for deployment. The modest source-only transfer results, especially the low hospital-to-PTB-XL sensitivity at the source-selected threshold, reinforce that additional calibration, adaptation, and prospective validation are necessary before any clinical decision-support use.

## Figures and Tables

**Table 1:** PTB-XL SCP statement codes used to define the MI class in the binary task.

Code	Type	Records	Meaning in the PTB-XL SCP Statement Table
ALMI	MI location statement	288	Anterolateral myocardial infarction
AMI	MI location statement	353	Anterior myocardial infarction
ASMI	MI location statement	2357	Anteroseptal myocardial infarction
ILMI	MI location statement	478	Inferolateral myocardial infarction
IMI	MI location statement	2676	Inferior myocardial infarction
INJAL	Injury statement mapped to MI	145	Subendocardial injury in anterolateral leads
INJAS	Injury statement mapped to MI	214	Subendocardial injury in anteroseptal leads
INJIL	Injury statement mapped to MI	15	Subendocardial injury in inferolateral leads
INJIN	Injury statement mapped to MI	18	Subendocardial injury in inferior leads
INJLA	Injury statement mapped to MI	17	Subendocardial injury in lateral leads
IPLMI	MI location statement	51	Inferoposterolateral myocardial infarction
IPMI	MI location statement	33	Inferoposterior myocardial infarction
LMI	MI location statement	201	Lateral myocardial infarction
PMI	MI location statement	17	Posterior myocardial infarction

Note: Counts show how many retained PTB-XL records contained each SCP code. The counts are not mutually exclusive, so one record can contribute to more than one row.

**Table 2:** Characteristics of the two datasets used in the main study.

Dataset	Records	Female/Male	Patients	Healthy	MI	MI (%)	Age Summary
Hospital dataset	1749	666/1083	1434	1550	199	11.38	mean 66.82 ± 28.74; median 62
PTB-XL binary subset	14,982	7793/7189	13,436	9513	5469	36.50	mean 59.64 ± 29.70; median 59

Note: The counts are reported at record level. Age summaries are descriptive metadata only and were not used for model training.

**Table 3:** Feature families used in the study.

Feature Family	Main Signal Emphasis	Summary
Beat-template morphology	Morphology	Beat-template amplitudes, waveform areas, and representative beat-shape measures for each lead.
Repolarization descriptors	T-wave/repolarization	Lead-wise T-wave amplitude, area, polarity, symmetry, and related repolarization summaries.
Lead-group spatial descriptors	Multi-lead spatial relations	Group-level summaries across anterior, inferior, lateral, limb, and precordial leads.
Fiducial interval descriptors	Intervals	Clinically meaningful interval and segment-duration measures derived from beat templates.
Custom ST descriptors	ST segment	ST features from the project-specific detector under the shared fallback rule.
NeuroKit ST descriptors	ST segment	ST features derived from the NeuroKit-based delineation under the shared fallback rule.
Dual ST descriptor bank	ST segment	Combined custom and NeuroKit ST descriptors with the same fallback policy.
Compact clinical feature bank	Hybrid	Curated amplitude, morphology, interval, and repolarization descriptors.
Broad hybrid feature bank	Hybrid	Non-ST hybrid feature set combining morphology, repolarization, interval, and spatial information.
Broad hybrid + ST bank	Hybrid + ST	Broad hybrid feature set augmented with ST information.
Lead-connectivity descriptors	Connectivity	Lead-to-lead consistency and multivariate relation descriptors built from signal and template behavior.

**Table 4:** Hospital imbalance-policy selection experiment. The mild random-oversampling policy was retained for hospital-trained analyses because it gave the highest mean balanced accuracy while keeping the Brier score below the class-weight-only alternative.

Policy	Mean Balanced Accuracy (BA)	95% Confidence Interval (CI)	Mean Sensitivity	Mean Brier
No balancing	0.729	0.723–0.736	0.678	0.0865
Class weighting only	0.726	0.720–0.733	0.691	0.1047
Mild oversampling	0.731	0.724–0.737	0.693	0.0917
Moderate oversampling	0.728	0.722–0.735	0.685	0.0971

**Table 5:** Top internal results in each dataset. Only the main threshold-tuned test metrics are shown in the table body; confusion counts are reported in the text.

Dataset	Rank	Feature Family	Model	BA	ROC-AUC	PR-AUC	Sens.	Spec.	Thr.
Hospital	1	Fiducial interval descriptors	Extra Trees	0.775	0.855	0.479	0.724	0.827	0.205
Hospital	2	Dual ST descriptor bank	Extra Trees	0.763	0.841	0.412	0.754	0.772	0.170
Hospital	3	Broad hybrid + ST bank	Extra Trees	0.762	0.840	0.437	0.734	0.790	0.160
PTB-XL	1	Broad hybrid + ST bank	XGBoost	0.898	0.965	0.953	0.842	0.953	0.375
PTB-XL	2	Broad hybrid feature bank	XGBoost	0.893	0.966	0.954	0.824	0.962	0.475
PTB-XL	3	Beat-template morphology	XGBoost	0.890	0.959	0.946	0.849	0.931	0.325

**Table 6:** Structured interpretation of candidate contributors to directional transfer asymmetry.

Candidate Contributor	Evidence in This Study	Interpretation
Sample size and prevalence	PTB-XL provided many more training records and a higher MI prevalence than the hospital source.	These differences may affect learned probability scale, threshold selection, and stability of feature–label associations. A size-matched PTB-XL down-sampling sensitivity analysis is therefore useful for separating sample-size effects from other dataset-shift effects.
Label definition and disease stage	Hospital MI labels came from a chest-pain clinical workflow, whereas PTB-XL MI-superclass SCP codes include infarction-location and injury-related statements.	The two binary labels are harmonized for benchmarking but should not be assumed to be identical clinical constructs.
Acquisition and device conditions	Hospital ECGs came mainly from one institution and one acquisition system, whereas PTB-XL is a public benchmark with its own acquisition pipeline.	Device, preprocessing, and waveform-distribution differences may shift amplitude- and interval-based features even after common filtering and mV scaling.
Feature-distribution shift	The leading feature family changed across internal and transfer settings.	This supports a feature-shift explanation and motivates reporting aggregate ST-amplitude, QRS-duration, and missingness summaries without releasing private records.
Threshold transportability	Hospital-to-PTB-XL transfer retained ROC-AUC 0.774 but had sensitivity 0.164 at the source-selected threshold.	Ranking ability and operating-threshold validity can diverge; threshold calibration is a distinct external-validation requirement.

**Table 7:** Top strict source-only cross-dataset transfer results in each direction. All model selection and threshold selection used source-dataset labels only.

Direction	Rank	Features	Model	BA	ROC-AUC	PR-AUC	Sens.	Spec.	Thr.
Hospital → PTB-XL	1	Repolarization descriptors	Logistic reg.	0.580	0.774	0.726	0.164	0.996	0.150
Hospital → PTB-XL	2	Lead-connectivity descriptors	SVM	0.576	0.599	0.505	0.300	0.852	0.250
Hospital → PTB-XL	3	Broad hybrid feature bank	Logistic reg.	0.565	0.605	0.529	0.165	0.964	0.125
PTB-XL → hospital	1	Lead-connectivity descriptors	Logistic reg.	0.632	0.635	0.161	0.553	0.710	0.450
PTB-XL → hospital	2	Beat-template morphology	Logistic reg.	0.615	0.619	0.166	0.384	0.847	0.525
PTB-XL → hospital	3	Repolarization descriptors	XGBoost	0.610	0.624	0.212	0.610	0.610	0.450

Note: Rows are ranked within a prespecified feature-family by model-family benchmark grid. They should not be interpreted as validation of one prespecified final model.

**Table 8:** Best strict source-only transfer result versus best secondary target-threshold selection result in each direction.

Direction	Strict Feature Family	Strict Model	Strict BA	Threshold-Selected Feature Family	Model	Threshold-Selected BA	ΔBA
Hospital → PTB-XL	Repolarization descriptors	Logistic reg.	0.580	Repolarization descriptors	Logistic reg.	0.682	+0.102
PTB-XL → hospital	Lead-connectivity descriptors	Logistic reg.	0.632	Broad hybrid + ST bank	XGBoost	0.640	+0.009

Note: Threshold-selected BA denotes balanced accuracy after target-label threshold re-selection. This is a secondary analysis and is not a strict source-only transfer result.

**Table 9:** Position of the present study relative to the three most similar ECG-MI papers.

Study	Main Data and Task	Model/Validation Emphasis	Relevance to the Present Paper
Liu et al. (EvoMBN) [23]	PTB MI detection/localization with PTB-XL architecture transfer.	Genetic-algorithm architecture optimization, multi-branch 12-lead processing, and lead squeeze-and-excitation weighting.	Shows the importance of inter-patient evaluation, lead-aware modeling, and cross-database transfer, but the focus is neural architecture transfer rather than fixed engineered-feature transportability.
Rai et al. [24]	PTB-XL, public ECG-image data, and hospital paper ECG for MI/non-MI and rhythm classification.	Automated paper-ECG digitization, DWT/CWT preprocessing, hybrid CNN–RNN models, and training-retraining on diverse sources.	Supports the need to discuss input-format and hospital-data shift; its retraining design is related to adaptation, not strict source-only external validation.
Bao et al. [25]	PTB-XL and a clinical CPC dataset for MI detection/localization.	Lightweight single-lead 1D-CNN–CBAM–BiLSTM detection, SHAP-guided three-lead localization, transfer learning, and Grad-CAM/SHAP interpretation.	Reinforces the value of lead importance and clinical feature structure; its transfer learning results should not be numerically compared with source-only transfer in this paper.
Present study	Cardiologist-annotated hospital data and PTB-XL for binary healthy-versus-MI classification.	Eleven engineered feature families and five classifier families under matched preprocessing, patient-aware splitting, source-only transfer, and secondary target-threshold analysis.	Provides a transparent benchmark of how much engineered ECG feature performance remains when the dataset changes without target-domain retraining or fine-tuning.

## Data Availability

PTB-XL is publicly available as described by Wagner et al. [34]. The private hospital dataset is not publicly available because it contains institution-controlled clinical ECG data and privacy restrictions. De-identified aggregate result tables, non-sensitive configuration summaries, and selected code pieces for specific analysis functionalities can be made available from the corresponding author upon reasonable request and after institutional review. The complete hospital software cannot be released as a runnable public package because it was developed for government-hospital workflows, depends on institution-specific ECG data structures and access controls, and cannot be executed correctly without restricted hospital data.

## Supplementary Materials

The following supporting information can be downloaded at https://www.mdpi.com/article/10.3390/diagnostics16132061/s1, Table S1, complete internal, strict source-only transfer, and secondary target-threshold result exports; Table S2, feature-family definitions, dimensions, and missingness summaries; Table S3, merged hyperparameter-search and threshold-selection policy; Table S4, ST quality-control and fallback counts; and Table S5, strict source-only versus secondary target-threshold results.

## Abbreviations

The following abbreviations are used in this manuscript:

aECG	Annotated electrocardiogram
AI	Artificial intelligence
AUC	Area under the curve
BA	Balanced accuracy
CBAM	Convolutional block attention module
CI	Confidence interval
CNN	Convolutional neural network
CPC	Chest Pain Center
CWT	Continuous wavelet transform
DWT	Discrete wavelet transform
ECG	Electrocardiogram
Grad-CAM	Gradient-weighted class activation mapping
HL7	Health Level Seven
LG-MSL	Lead-grouped multi-stage learning
MI	Myocardial infarction
OMI	Occlusion myocardial infarction
PR-AUC	Area under the precision–recall curve
PTB-XL	PTB-XL electrocardiography dataset
QC	Quality control
ROC-AUC	Area under the receiver operating characteristic curve
SCP	Standard communications protocol for computer-assisted electrocardiography
SHAP	Shapley additive explanations
ST	ST segment
STEMI	ST-segment elevation myocardial infarction
SVM	Support vector machine
TUSEB	Health Institutes of Türkiye
WFDB	WaveForm Database
XML	Extensible markup language
